# Clusterin serum levels are elevated in patients with early rheumatoid arthritis and predict disease activity and treatment response

**DOI:** 10.1038/s41598-021-90973-2

**Published:** 2021-06-01

**Authors:** Tereza Kropáčková, Heřman Mann, Olga Růžičková, Olga Šléglová, Lucia Vernerová, Veronika Horváthová, Michal Tomčík, Karel Pavelka, Jiří Vencovský, Ladislav Šenolt

**Affiliations:** 1 Institute of Rheumatology, Na Slupi 4, 128 00 Prague 2, Czech Republic; 2 Department of Rheumatology, 1st Faculty of Medicine, Charles University, Prague, Czech Republic; 3 Faculty of Science, Charles University, Prague, Czech Republic

**Keywords:** Rheumatology, Rheumatic diseases

## Abstract

Clusterin (CLU) is a molecular chaperone that participates in a variety of biological processes. Recent studies indicate its possible involvement in the development of bone erosions and autoimmunity. The aim of this study was to investigate its serum concentrations in patients with early rheumatoid arthritis (RA) and to explore their potential relationship with disease activity and treatment response. Serum levels of CLU were measured in 52 patients before and 3 months after the initiation of treatment and in 52 healthy individuals. CLU levels at baseline were significantly increased in patients with early RA compared with healthy subjects (p < 0.0001). After 3 months of treatment, the levels of CLU decreased and reached concentrations comparable to those in controls. Even though there was no relationship between CLU levels and disease activity at baseline, CLU levels positively correlated with disease activity at months 3, 6 and 12 after treatment initiation. Using ROC analysis, lower CLU baseline levels predicted achieving the therapeutic target of low disease activity and remission at months 3, 6 and 12. In summary, we found increased serum concentrations of clusterin in treatment-naïve patients with early rheumatoid arthritis, and we suggest clusterin as a predictive biomarker of disease activity and treatment response.

## Introduction

Rheumatoid arthritis (RA) is a chronic autoimmune disorder characterized by synovial membrane inflammation that leads to pain, swelling and structural damage of the affected joints as well as reduced physical function and quality of life^1^. In recent years, considerable progress has been made in understanding the pathophysiological processes in RA, which has enabled the development of new treatment strategies that have subsequently provided further insight into the pathogenic mechanisms of the disease^2,3^. However, despite the large number of therapeutic agents currently available for RA, some patients still do not achieve the therapeutic target of low disease activity or remission^4^. The identification of a reliable predictive biomarker of RA remains still a challenge. To date, several candidate biomarkers for the prediction of disease activity and for distinguishing between responders and non-responders have been studied. However, their potential utilization in clinical practice needs to be confirmed in larger cohorts^5–7^.

Clusterin (CLU) is a secreted heterodimeric glycoprotein with a molecular mass of 80 kD. It consists of two distinct polypeptide chains linked by disulfide bonds^8,9^. The expression of CLU has been demonstrated in a wide variety of tissues, especially at the sites of fluid-tissue interfaces^10^, and its dysregulation is associated with diverse disease states, including cancer^11^, Crohn’s disease^12^, osteoarthritis (OA)^13^ and RA^14^. CLU exerts a chaperone-like activity similar to that of small heat shock proteins^15^, and it has cytoprotective and anti-apoptotic properties^16,17^. Moreover, CLU inhibits the enzymatic activity of matrix metalloproteinase (MMP)-2, MMP-3, MMP-7 and MMP-9^18^. CLU is also able to reduce the formation of osteoclasts by inhibiting the macrophage colony-stimulating factor (M-CSF)-induced activation of extracellular signal-regulated kinase (ERK) and could thereby reduce the development of bone erosions^19^. In our previous study, serum concentrations of CLU were found to be lower in patients with hand OA, especially in those with erosive disease, than in healthy individuals^20^. Recently, CLU has been suggested to exhibit a protective function in inflammation and autoimmune diseases^21^.

In synovial tissue, CLU is predominantly produced by synovial lining cells and is expressed at lower levels in RA than in OA. However, the concentrations of CLU in synovial fluid are comparable between RA and OA^14^. The downregulation of CLU gene in cultured RA synoviocytes increased the production of interleukin (IL)-6 and IL-8^14^. On the other hand, the transfection of CLU to synoviocytes resulted in the inhibition of tumour necrosis factor (TNF)-induced activation of nuclear factor (NF)-κB (a key transcriptional regulator of IL-6 and IL-8)^22^. These data suggest an involvement of CLU in the pathophysiology of inflammatory diseases such as arthritis, with a potentially protective role. However, the abovementioned properties were attributed to intracellular form of CLU and circulating levels of secretory CLU (sCLU) were not investigated. Moreover, there are no studies on the ability of CLU to reflect or predict disease activity. Therefore, the aim of our study was to analyse the serum levels of CLU in patients with early RA and in healthy individuals and to examine their potential association with disease activity and treatment response.

## Methods

### Characteristics of patients

A total of 52 patients with treatment-naïve early RA were included in this study. The patients fulfilled the 2010 American College of Rheumatology (ACR)/European League Against Rheumatism (EULAR) classification criteria for RA^23^, with a duration of symptoms < 6 months, and were prospectively followed in the Prague Early RA Clinic (PERAC) at the Institute of Rheumatology in Prague, Czech Republic. The control group consisted of 52 age-/sex-matched healthy individuals with no history of rheumatic and autoimmune disorder, cancer or severe chronic infectious disease. Written informed consent from each subject was obtained prior to study initiation, and the study was approved by the local ethics committee.

The clinical examinations were performed by qualified rheumatologists. Disease activity was evaluated by the Clinical Disease Activity Index (CDAI), Simplified Disease Activity Index (SDAI) and 28-joint Disease Activity Score (DAS28) using the erythrocyte sedimentation rate (ESR), the number of tender and swollen joints and the patient’s global visual analogue scale (VAS) at baseline and at months 3, 6, and 12 after treatment initiation. Patients were treated with conventional synthetic disease-modifying antirheumatic drugs (csDMARDs) and glucocorticoids (GCs). The therapeutic target, achieving low disease activity or remission, was evaluated based on SDAI, CDAI and DAS28 criteria^24,25^. The treatment response was assessed using the EULAR response criteria or as a relative SDAI/CDAI change from baseline^24,26^.

### Laboratory measurements

The serum CLU levels were measured in patients with early RA (prior to and after 3 months of therapy) and in healthy controls by an enzyme-linked immunosorbent assay (ELISA) according to the manufacturer’s instructions (BioVendor, Brno, Czech Republic). The limit of detection was 5 ng/ml, and the detection range of the assay was 5–160 ng/ml. The intra- and inter-assay coefficients of the variations were 6.2% and 7.8%, respectively. The absorbance was determined using a Sunrise ELISA reader (Tecan, Salzburg, Austria) with 450 nm as the primary wavelength.

C-reactive protein (CRP) levels were measured turbidimetrically using the Beckman Coulter AU system (Beckman Coulter, Brea, CA, USA). Anti-cyclic citrullinated peptide antibodies (anti-CCP) and IgM rheumatoid factor (RF) were analysed in serum by standard ELISA kits (TestLine, Brno, Czech Republic).

### Statistical analysis

The data are presented as the mean and the standard deviation (SD) unless stated otherwise. Statistical analyses were performed using GraphPad Prism 6 (GraphPad Software, San Diego, CA, USA). The normal distribution was determined by the D’Agostino and Pearson omnibus normality test. The unpaired t test or the Mann–Whitney test was performed for the comparison of continuous variables between two groups. For the comparison of CLU levels at baseline and at month 3 after treatment initiation in early RA patients, a paired t-test was used. The difference in the CLU levels between healthy controls and patients at baseline and at month 3 was assessed by one-way ANOVA. Spearman’s and Pearson’s correlation coefficients were calculated to evaluate the association between the CLU levels and other variables. Receiver operating characteristic (ROC) curve analysis of CLU levels was performed to predict disease activity and treatment response after 3, 6 and 12 months of treatment. The area under the curve (AUC) and the 95% confidence interval (CI) were calculated. P values less than 0.05 were considered statistically significant.

### Ethics approval and consent to participate

The study was approved by the local ethics committee of the Institute of Rheumatology in Prague, Czech Republic. Written informed consent was obtained from all participants. All methods were performed in accordance with relevant guidelines and regulations.

## Results

### Clinical characteristics

The baseline demographic and clinical characteristics of the subjects are summarized in Table 1. The patients included 16 males and 36 females with a mean age of 50.8 years. Anti-CCP positivity and RF positivity were found in 52% and 60% of patients, respectively. Therapy with csDMARDs was initiated in 51 patients: 43 patients were treated with methotrexate (MTX, mean 15 mg/week), 7 with sulfasalazine (2 g/day) and 1 with leflunomide (20 mg/day). Forty-six patients initially received combined therapy of csDMARDs with low-dose GCs (≤ 10 mg/day prednisone or equivalent). One patient was treated with GCs alone due to planned conception. The disease activity of the patients at baseline and at months 3, 6 and 12 after treatment initiation is shown in Table 2.

**Table 1:** Baseline characteristics of early RA patients and healthy controls.

	Early RA	Healthy controls
Patients, *n*	52	52
Males/females, *n* (%)	16/36 (30.8/69.2)	16/36 (30.8/69.2)
Age, *years*	50.8 ± 16.1	50.5 ± 15.3 (p = 0.926)
BMI, *kg/m*^*2*^	25.4 ± 4.6	26.2 ± 4.2 (p = 0.337)
CRP, *mg/l*	18.9 ± 24.0	2.7 ± 3.8 (p < 0.001)
ESR, *mm/1st hour*	34.0 ± 24.0	–
DAS28	5.5 ± 1.4	–
CDAI	30.6 ± 16.1	–
SDAI	32.5 ± 17.5	–
Disease duration,* months*	< 6	–
RF positivity, *n* (%)	31 (59.6)	–
Anti-CCP positivity, *n* (%)	27 (51.9)	–
**csDMARDs/GCs, *n* (%)**	51/47 (98.1/90.4)	–
Methotrexate, *n* (%)	43 (82.7)	–
Sulfasalazine, *n* (%)	7 (13.5)	–
Leflunomide, *n* (%)	1 (1.9)	–

*Anti-CCP* anti-cyclic citrullinated peptide antibodies, *BMI* body mass index, *CDAI* Clinical Disease Activity Index, *CRP* C-reactive protein, *csDMARDs* conventional synthetic disease-modifying antirheumatic drugs, *DAS28* 28-joint Disease Activity Score, *ESR* erythrocyte sedimentation rate, *GCs* glucocorticoids, *RA* rheumatoid arthritis, *RF* rheumatoid factor, *SDAI* Simplified Disease Activity Index. Data are presented as the mean ± the SD.

**Table 2:** Disease activity of the early RA patients.

	Baseline	Month 3	Month 6	Month 12
**DAS28**				
High (> 5.1), *n* (%)	32 (61.5)	6 (11.5)	5 (9.6)	3 (5.8)
Moderate (> 3.2 and ≤ 5.1), *n* (%)	19 (36.5)	10 (19.2)	14 (26.9)	14 (26.9)
Low (> 2.6 and ≤ 3.2), *n* (%)	1 (1.9)	10 (19.2)	9 (17.3)	7 (13.5)
Remission (≤ 2.6), *n* (%)	0 (0.0)	26 (50.0)	24 (46.2)	28 (53.8)
**CDAI**				
High (> 22), *n* (%)	30 (57.7)	6 (11.5)	7 (13.5)	3 (5.8)
Moderate (> 10 and ≤ 22), *n* (%)	20 (38.5)	6 (11.5)	8 (15.4)	7 (13.5)
Low (> 2.8 and ≤ 10), *n* (%)	2 (3.8)	19 (36.5)	15 (28.8)	21 (40.4)
Remission (≤ 2.8), *n* (%)	0 (0.0)	21 (40.4)	22 (42.3)	21 (40.4)
**SDAI**				
High (> 26), *n* (%)	26 (50.0)	4 (7.7)	5 (9.6)	2 (3.8)
Moderate (> 11 and ≤ 26), *n* (%)	24 (46.2)	8 (15.4)	7 (13.5)	7 (13.5)
Low (> 3.3 and ≤ 11), *n* (%)	2 (3.8)	18 (34.6)	17 (32.7)	23 (44.2)
Remission (≤ 3.3), *n* (%)	0 (0.0)	22 (42.3)	23 (44.2)	20 (38.5)

*CDAI* Clinical Disease Activity Index, *DAS28* 28-joint Disease Activity Score, *SDAI* Simplified Disease Activity Index.

### Clusterin levels are higher in patients with early RA than in healthy controls

**Figure 1:**
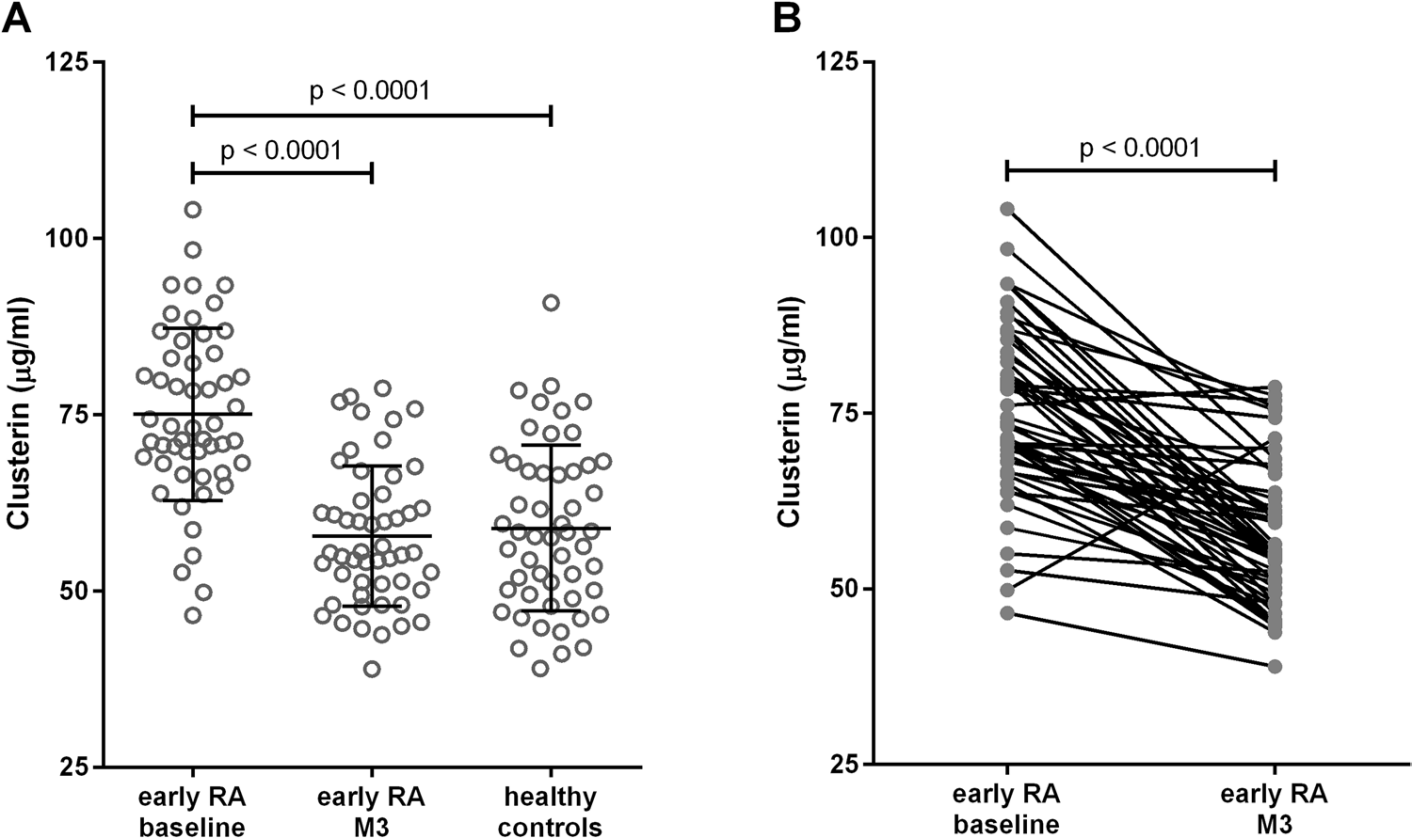
Serum clusterin levels in patients with early rheumatoid arthritis (RA) at baseline and 3 months after the initiation of treatment (M3) and in healthy individuals (**A**). Comparison of changes in individual patients before and after 3 months of therapy (**B**). The horizontal lines represent the mean ± the SD.

Baseline CLU concentrations were significantly higher in patients with treatment-naïve early RA than in healthy individuals (75.1 ± 12.2 vs 58.9 ± 11.7 µg/ml, p < 0.0001). After 3 months of therapy, CLU levels in patients significantly decreased (75.1 ± 12.2 vs 57.8 ± 9.9 µg/ml, p < 0.0001) and reached levels comparable to those in healthy subjects (57.8 ± 9.9 vs 58.9 ± 11.7 µg/ml, p = 0.865) (Fig. 1). Overall, only two patients showed an elevation in the CLU levels, while the other 50 showed a decrease after 3 months of treatment (Fig. 1). There were no significant associations between the baseline CLU concentrations and sex, age, body mass index (BMI), CRP, ESR, GCs dose equivalent to prednisone, MTX dose, RF or anti-CCP.

### Clusterin levels predict disease activity and treatment response in early RA patients

**Figure 2:**
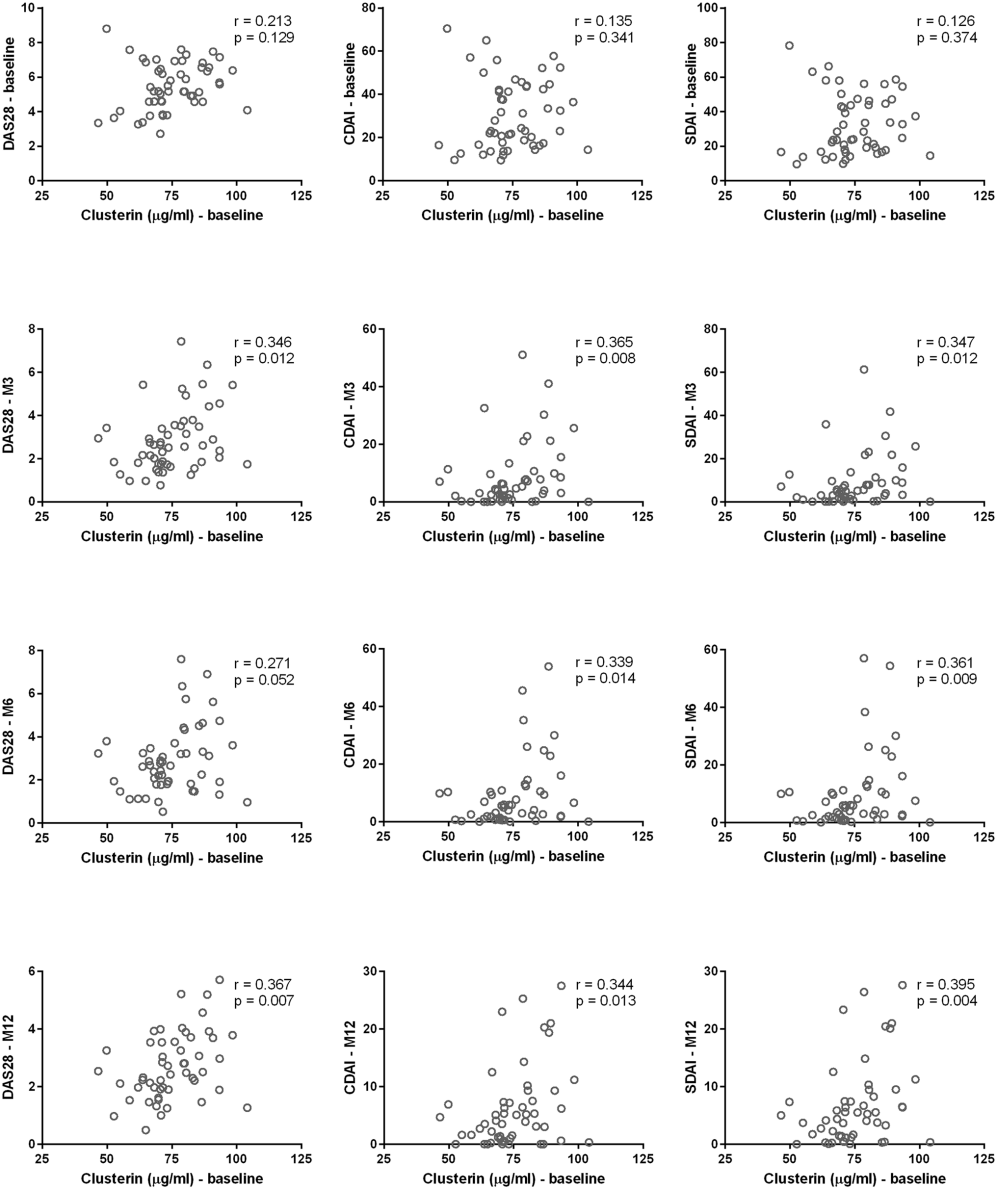
Correlations between baseline clusterin levels and disease activity according to the CDAI, SDAI and DAS28 at baseline and months 3 (M3), 6 (M6) and 12 (M12) after treatment initiation.

Although there was no association between CLU levels and disease activity at baseline, CLU baseline levels were positively correlated with the CDAI and SDAI at month 3 (r = 0.365, p = 0.008; r = 0.347, p = 0.012, respectively), month 6 (r = 0.339, p = 0.014; r = 0.361, p = 0.009, respectively) and month 12 (r = 0.344, p = 0.013; r = 0.395, p = 0.004, respectively) after treatment initiation (Fig. 2). Significant positive correlations were also found between baseline CLU levels and the DAS28 at months 3 and 12 (r = 0.346, p = 0.012; r = 0.367, p = 0.007, respectively); however, the correlation at month 6 showed only a nonsignificant trend towards a positive association (r = 0.271, p = 0.052) (Fig. 2). The change in the CLU levels from baseline to month 3 did not correlate with disease activity or its change at any time point.

**Figure 3:**
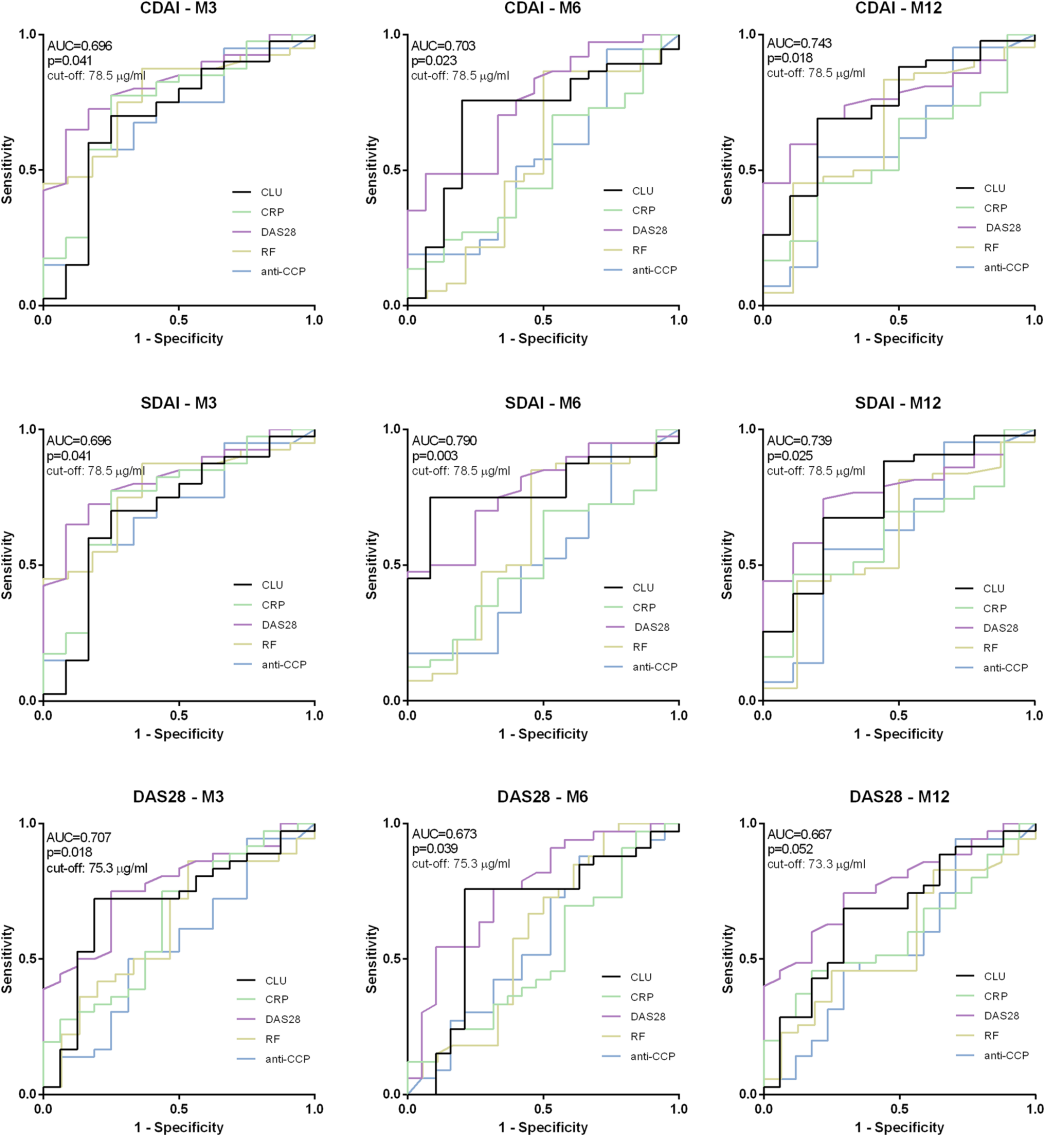
ROC curve analysis of baseline CLU levels, DAS28, RF, anti-CCP and CRP levels for prediction of achieving low disease activity or remission after 3, 6 and 12 months of treatment according to the CDAI, SDAI and DAS28.

Using ROC curve analysis, lower CLU baseline levels predicted achieving remission and low disease activity according to the CDAI and SDAI at months 3 [AUC = 0.696 (95% CI 0.511; 0.881), p = 0.041 for both], 6 [AUC = 0.703 (95% CI 0.540; 0.865), p = 0.023; AUC 0.790 (95% CI 0.665; 0.914), p = 0.003, respectively] and 12 [AUC = 0.743 (95% CI 0.578; 0.908), p = 0.018; AUC = 0.739 (95% CI 0.559; 919), p = 0.025, respectively] and at months 3 and 6 according to the DAS28 [AUC = 0.707 (95% CI 0.549; 0.864), p = 0.018; AUC = 0.673 (95% CI 0.506; 0.840), p = 0.039, respectively]. The ROC curve analysis for month 12 did not reach statistical significance [AUC = 0.667 (95% CI 0.509; 0.826), p = 0.052] (Fig. 3).

**Figure 4:**
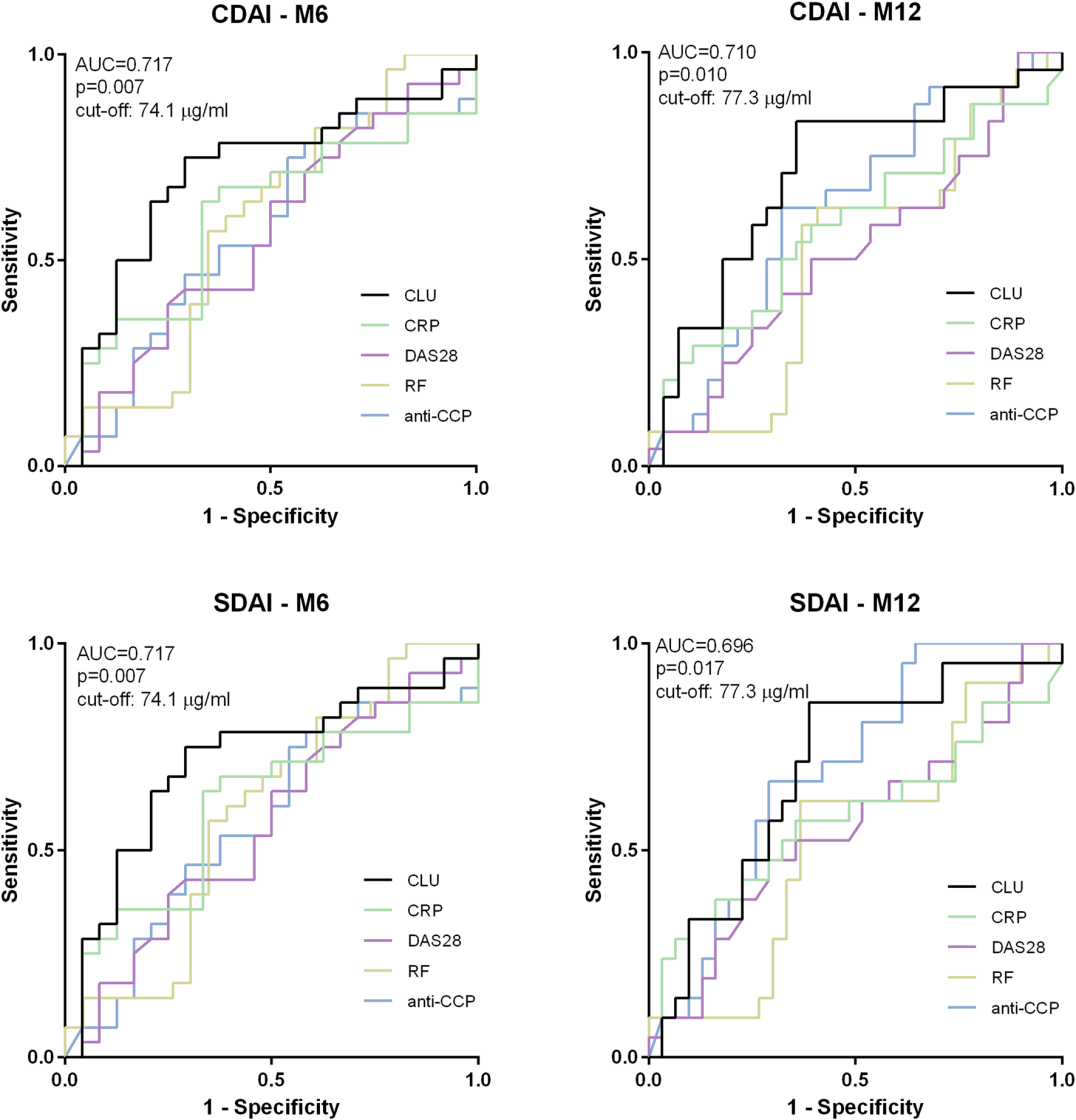
ROC curve analysis of baseline CLU levels, DAS28, RF, anti-CCP and CRP levels for prediction of achieving major treatment response after 6 and 12 months of treatment according to the CDAI and SDAI.

In addition, lower CLU baseline levels predicted major treatment response (≥ 85% relative improvement) based on the CDAI and SDAI after 6 [AUC = 0.717 (95% CI 0.573; 0.862), p = 0.007 for both] and 12 [AUC = 0.710 (95% CI 0.564; 0.856), p = 0.010; AUC = 0.696 (95% CI 0.548; 0.843), p = 0.017, respectively] months of therapy (Fig. 4). The ability of CLU baseline levels to predict good response by the DAS28 (improvement > 1.2 and current DAS28 ≤ 3.2) was not found (data not shown).

For the comparison of the predictive value of the baseline CLU levels with clinically used markers, we performed ROC curve analyses of baseline DAS28, anti-CCP, RF and CRP levels (Supplementary Table S1). Lower baseline DAS28 predicted achieving remission and low disease activity at all studied time points according to the DAS28 (p = 0.002 at months 3, 6 and 12), CDAI (p < 0.001 at month 3; p = 0.004 at month 6; p = 0.011 at month 12) and SDAI (p < 0.001 at month 3; p = 0.004 at month 6; p = 0.012 at month 12). ROC analyses of the baseline CRP and RF were statistically significant only for the prediction of remission and low disease activity according to the CDAI and SDAI at month 3 (CRP: p = 0.012 for both; RF: p = 0.005 for both). Baseline anti-CCP did not predict the achievement of the therapeutic target at any time point. ROC analyses for the prediction of achieving major treatment response according to the CDAI and SDAI did not reach statistical significance except for baseline anti-CCP levels according to the SDAI at month 12 (Supplementary Table S1).

## Discussion

In the present study, we examined the ability of CLU to predict disease activity and treatment response in treatment-naïve early RA patients. We demonstrated increased CLU levels in treatment-naïve patients with early RA and their decrease after the initiation of conventional therapy. In addition, baseline CLU levels predicted achieving the therapeutic target of low disease activity and remission during the first year.

Evidence suggests that CLU exerts a cytoprotective function under stress conditions, mediated, for example, by protection against oxidative stress or inhibition of apoptosis and inflammation^27^, which are all involved in the pathogenesis of RA. However, to our knowledge, there is no study on circulating levels of CLU in RA patients and their potential use as a biomarker. We found higher serum concentrations of CLU in patients with early RA compared to healthy controls. Previous findings in RA by Devauchelle et al.^14^ reported no difference in the CLU levels in synovial fluid between OA and RA, although they presented lower mRNA expression in RA synovial tissue. There are several possible explanations why circulating levels do not correspond with the previous findings from the joint.

First, CLU is ubiquitously expressed in most cells and tissues^10,28^, and is upregulated under a variety of pathological conditions including ageing, diabetes, atherosclerosis and degenerative diseases^29,30^. Therefore, apart from affected joints, systemic manifestations of RA may significantly contribute to circulating levels of CLU. For instance, patients with RA have increased risk of heart disease and their vasculature is influenced by systemic inflammation^31^. CLU is expressed in vascular endothelial cells as well as in other components of the circulatory system and sites of vascular disease or injury^32^. During vascular damage, sCLU was found to accumulate in the human serum of diabetes type II patients^33^ or during myocardial infarction^32^. CLU is also significantly related to the most atherogenic components of lipid profile (total cholesterol and LDL), especially in women^34^. Patients with RA tend to have a different profile of cardiac risk factors, including a higher frequency of smoking and an altered lipid profile (unfavourable ratio of total to HDL cholesterol)^31^.

Second, CLU protein exists in several isoforms that differ in their localization and function. The predominant form is a secretory CLU that is glycosylated and secreted into the extracellular space as a heterodimeric protein with a molecular mass of approximately 75–80 kDa^35^. Other and rarer isoforms of the protein are localized in the cytosol or nucleus^36,37^. The secreted form of CLU has been shown to be cytoprotective^16^, whereas the nuclear form is proapoptotic^36^. The study showing reduced CLU expression in RA synovium^14^ analysed the levels of the 40- to 50-kDa forms, which are the major intracellular forms of CLU. Apart from different proportion of individual CLU isoforms in the circulation, cells and tissues we can also assume unequal changes in their expression based on different stimuli and response to treatment. Moreover, it is also important to note that changes in mRNA expression do not always correspond with the changes in the protein levels.

Third, unlike previous studies, all patients in our study were treatment-naïve, with a short duration of disease symptoms. It can be suggested that CLU protein levels can change over the years of the disease as a result of effective long-term therapy. For instance, DMARDs/GCs treatment has been found to be associated with a marked reduction in synovial tissue macrophage infiltration^38,39^. This change in synovial inflammatory condition during treatment can possibly be reflected by a decrease in CLU after 3 months of treatment to levels comparable to healthy individuals, observed in our study.

RA is a heterogeneous disease, and patients differ in the severity of symptoms as well as in clinical and laboratory findings. In addition, the efficacy of initial therapy using csDMARDs and GCs varies among different individuals. Therefore, finding a suitable biomarker for the prediction of treatment response could help tailor first-choice therapeutic agents in potential non-responders to conventional therapy. Early RA diagnosis together with the prompt initiation of effective treatment may facilitate the achievement of rapid disease remission and the prevention of structural changes, disability and negative impacts on patients’ quality of life. In the present study, we found that lower CLU levels at baseline predicted achieving the therapeutic target of low disease activity and remission after 3, 6 and 12 months of treatment. Moreover, lower baseline CLU levels predicted ≥ 85% improvement after 6 and 12 months of therapy. Consequently, CLU could serve as a biomarker for the prediction of disease activity and treatment response in treatment-naïve patients with early RA. In addition, CLU performed much better than CRP, a routinely used marker of inflammation, in predicting disease activity and treatment response.

Furthermore, CLU was also better than autoantibodies to predict treatment response. Importantly, although DAS28 was a slightly better predictor than CLU at certain time points predicting low disease activity and remission, CLU was demonstrated as a better biomarker of major treatment response, which is an important therapeutic target in clinical practice.

However, further studies using larger cohorts of patients are required to demonstrate whether CLU is also able to predict structural disease progression and the therapeutic response in patients with established RA to other treatments, e.g. targeted DMARDs.

## Conclusions

In summary, we have demonstrated increased CLU levels in treatment-naïve patients with early RA in comparison to healthy individuals and their decrease after the initiation of conventional therapy. In addition, CLU levels at baseline predicted achieving the therapeutic target of low disease activity and remission or major clinical response. These data suggest that CLU may serve as a potential predictive biomarker in patients with early RA.

## Supplementary Information

Supplementary Information 1.

## Data Availability

The data underlying this article will be shared on reasonable request to the corresponding author.

## Abbreviations

ACR: American College of Rheumatology
Anti-CCP: Anti-cyclic citrullinated peptide antibodies
AUC: Area under the curve
BMI: Body mass index
CDAI: Clinical disease activity index
CI: Confidence interval
CLU: Clusterin
CRP: C-reactive protein
csDMARDS: Conventional synthetic disease-modifying antirheumatic drugs
DAS28: 28-Joint disease activity score
ELISA: Enzyme-linked immunosorbent assay
ERK: Extracellular signal-regulated kinase
ESR: Erythrocyte sedimentation rate
EULAR: European League Against Rheumatism
GCs: Glucocorticoids
IL: Interleukin
M-CSF: Macrophage colony-stimulating factor
MMP: Matrix metalloproteinase
MTX: Methotrexate
NF: Nuclear factor
OA: Osteoarthritis
RA: Rheumatoid arthritis
RF: Rheumatoid factor
ROC: Receiver operating characteristic
sCLU: Secretory clusterin
SD: Standard deviation
SDAI: Simplified disease activity index
TNF: Tumour necrosis factor
VAS: Visual analogue scale
