# Supplementary material for: Clusterin serum levels are elevated in patients with early rheumatoid arthritis and predict disease activity and treatment response
Source: Sci Rep. 2021 Jun 1;11:11525. doi: 10.1038/s41598-021-90973-2 (PMC8169772; doi:10.1038/s41598-021-90973-2)
Supplement: Supplementary file 1 — Supplementary Information 1. [file 41598_2021_90973_MOESM1_ESM.pdf]

Supplementary Table S1. ROC analyses for the prediction of low disease activity and remission (**A**) and major treatment response (**B**) according to SDAI, CDAI and DAS28 at month 3 (M3), 6 (M6) and 12 (M12)

A. Prediction of low disease activity and remission

ROC analysis of baseline CLU levels

DAS28-M3	Results
Area under the ROC curve	0.7066
Std. Error	0.0805
95% confidence interval	0.5488 to 0.8644
P value	0.0183

DAS28-M6	Results
Area under the ROC curve	0.673
Std. Error	0.08509
95% confidence interval	0.5062 to 0.8399
P value	0.0392

DAS28-M12	Results
Area under the ROC curve	0.6672
Std. Error	0.08081
95% confidence interval	0.5088 to 0.8256
P value	0.0523

CDAI-M3	Results
Area under the ROC curve	0.6958
Std. Error	0.09444
95% confidence interval	0.5107 to 0.8810
P value	0.0412

CDAI-M6	Results
Area under the ROC curve	0.7027
Std. Error	0.08278
95% confidence interval	0.5404 to 0.8650
P value	0.0231

CDAI-M12	Results
Area under the ROC curve	0.7429
Std. Error	0.08429
95% confidence interval	0.5776 to 0.9081
P value	0.0179

SDAI-M3	Results
Area under the ROC curve	0.6958
Std. Error	0.09444
95% confidence interval	0.5107 to 0.8810
P value	0.0412

SDAI-M6	Results
Area under the ROC curve	0.7896
Std. Error	0.06351
95% confidence interval	0.6651 to 0.9141
P value	0.0025

SDAI-M12	Results
Area under the ROC curve	0.739
Std. Error	0.09167
95% confidence interval	0.5593 to 0.9187
P value	0.0253

ROC analysis of baseline DAS28

DAS28-M3	Results
Area under the ROC curve	0.7682
Std. Error	0.06656
95% confidence interval	0.6377 to 0.8987
P value	0.0022

DAS28-M6	Results
Area under the ROC curve	0.7608
Std. Error	0.07066
95% confidence interval	0.6222 to 0.8993
P value	0.0019

DAS28-M12	Results
Area under the ROC curve	0.7689
Std. Error	0.06498
95% confidence interval	0.6415 to 0.8963
P value	0.0018

CDAI-M3	Results
Area under the ROC curve	0.8198
Std. Error	0.061
95% confidence interval	0.7002 to 0.9394
P value	0.0009

CDAI-M6	Results
Area under the ROC curve	0.755
Std. Error	0.07192
95% confidence interval	0.6140 to 0.8959
P value	0.0043

CDAI-M12	Results
Area under the ROC curve	0.7595
Std. Error	0.06839
95% confidence interval	0.6255 to 0.8936
P value	0.0114

SDAI-M3	Results
Area under the ROC curve	0.8198
Std. Error	0.061
95% confidence interval	0.7002 to 0.9394
P value	0.0009

SDAI-M6	Results
Area under the ROC curve	0.7792
Std. Error	0.0689
95% confidence interval	0.6441 to 0.9142
P value	0.0036

SDAI-M12	Results
Area under the ROC curve	0.7674
Std. Error	0.07095
95% confidence interval	0.6283 to 0.9065
P value	0.0123

ROC analysis of baseline CRP levels

DAS28-M3	Results
Area under the ROC curve	0.6441
Std. Error	0.08422
95% confidence interval	0.4790 to 0.8092
P value	0.0998

DAS28-M6	Results
Area under the ROC curve	0.5152
Std. Error	0.08537
95% confidence interval	0.3478 to 0.6825
P value	0.8567

DAS28-M12	Results
Area under the ROC curve	0.5966
Std. Error	0.07935
95% confidence interval	0.4411 to 0.7522
P value	0.262

CDAI-M3	Results
Area under the ROC curve	0.7417
Std. Error	0.08528
95% confidence interval	0.5745 to 0.9088
P value	0.0118

CDAI-M6	Results
Area under the ROC curve	0.5243
Std. Error	0.08842
95% confidence interval	0.3510 to 0.6977
P value	0.7851

CDAI-M12	Results
Area under the ROC curve	0.5714
Std. Error	0.09328
95% confidence interval	0.3885 to 0.7543
P value	0.4861

SDAI-M3	Results
Area under the ROC curve	0.7417
Std. Error	0.08528
95% confidence interval	0.5745 to 0.9088
P value	0.0118

SDAI-M6	Results
Area under the ROC curve	0.5313
Std. Error	0.09283
95% confidence interval	0.3493 to 0.7132
P value	0.7446

SDAI-M12	Results
Area under the ROC curve	0.615
Std. Error	0.09263
95% confidence interval	0.4334 to 0.7966
P value	0.2818

ROC analysis of baseline RF

DAS28-M3	Results
Area under the ROC curve	0.6222
Std. Error	0.08717
95% confidence interval	0.4513 to 0.7931
P value	0.1724

DAS28-M6	Results
Area under the ROC curve	0.5918
Std. Error	0.09183
95% confidence interval	0.4117 to 0.7718
P value	0.2827

DAS28-M12	Results
Area under the ROC curve	0.567
Std. Error	0.08576
95% confidence interval	0.3988 to 0.7351
P value	0.4465

CDAI-M3	Results
Area under the ROC curve	0.7761
Std. Error	0.07172
95% confidence interval	0.6355 to 0.9167
P value	0.0054

CDAI-M6	Results
Area under the ROC curve	0.5502
Std. Error	0.1026
95% confidence interval	0.3490 to 0.7514
P value	0.5832

CDAI-M12	Results
Area under the ROC curve	0.6548
Std. Error	0.1051
95% confidence interval	0.4487 to 0.8608
P value	0.1483

SDAI-M3	Results
Area under the ROC curve	0.7761
Std. Error	0.07172
95% confidence interval	0.6355 to 0.9167
P value	0.0054

SDAI-M6	Results
Area under the ROC curve	0.6091
Std. Error	0.1074
95% confidence interval	0.3984 to 0.8197
P value	0.2717

SDAI-M12	Results
Area under the ROC curve	0.6119
Std. Error	0.111
95% confidence interval	0.3944 to 0.8295
P value	0.3187

ROC analysis of baseline anti-CCP

DAS28-M3	Results
Area under the ROC curve	0.5503
Std. Error	0.09018
95% confidence interval	0.3736 to 0.7271
P value	0.5653

DAS28-M6	Results
Area under the ROC curve	0.5662
Std. Error	0.08629
95% confidence interval	0.3970 to 0.7354
P value	0.4303

DAS28-M12	Results
Area under the ROC curve	0.5479
Std. Error	0.08951
95% confidence interval	0.3724 to 0.7234
P value	0.5782

CDAI-M3	Results
Area under the ROC curve	0.6833
Std. Error	0.0905
95% confidence interval	0.5059 to 0.8607
P value	0.056

CDAI-M6	Results
Area under the ROC curve	0.5405
Std. Error	0.09118
95% confidence interval	0.3618 to 0.7193
P value	0.6495

CDAI-M12	Results
Area under the ROC curve	0.6095
Std. Error	0.1047
95% confidence interval	0.4042 to 0.8149
P value	0.2855

SDAI-M3	Results
Area under the ROC curve	0.6833
Std. Error	0.0905
95% confidence interval	0.5059 to 0.8607
P value	0.056

SDAI-M6	Results
Area under the ROC curve	0.5208
Std. Error	0.1
95% confidence interval	0.3247 to 0.7170
P value	0.8281

SDAI-M12	Results
Area under the ROC curve	0.6202
Std. Error	0.1132
95% confidence interval	0.3983 to 0.8421
P value	0.2607

B. Prediction of major treatment response (≥ 85% relative improvement)

ROC analysis of baseline CLU levels

CDAI-M3	Results
Area under the ROC curve	0.6015
Std. Error	0.08067
95% confidence interval	0.4434 to 0.7597
P value	0.2146

CDAI-M6	Results
Area under the ROC curve	0.7173
Std. Error	0.07372
95% confidence interval	0.5727 to 0.8618
P value	0.0074

CDAI-M12	Results
Area under the ROC curve	0.7098
Std. Error	0.0744
95% confidence interval	0.5640 to 0.8557
P value	0.0096

SDAI-M3	Results	SDAI-M6	Results	SDAI-M12	Results
Area under the ROC curve	0.5837	Area under the ROC curve	0.7173	Area under the ROC curve	0.6959
Std. Error	0.08107	Std. Error	0.07372	Std. Error	0.07523
95% confidence interval	0.4248 to 0.7427	95% confidence interval	0.5727 to 0.8618	95% confidence interval	0.5484 to 0.8433
P value	0.3094	P value	0.0074	P value	0.0174

ROC analysis of baseline DAS28

CDAI-M3	Results	CDAI-M6	Results	CDAI-M12	Results
Area under the ROC curve	0.5045	Area under the ROC curve	0.561	Area under the ROC curve	0.5179
Std. Error	0.08033	Std. Error	0.08092	Std. Error	0.08154
95% confidence interval	0.3471 to 0.6620	95% confidence interval	0.4024 to 0.7196	95% confidence interval	0.3580 to 0.6777
P value	0.9557	P value	0.4517	P value	0.8257

SDAI-M3	Results	SDAI-M6	Results	SDAI-M12	Results
Area under the ROC curve	0.5123	Area under the ROC curve	0.561	Area under the ROC curve	0.5553
Std. Error	0.0803	Std. Error	0.08092	Std. Error	0.08337
95% confidence interval	0.3549 to 0.6697	95% confidence interval	0.4024 to 0.7196	95% confidence interval	0.3919 to 0.7187
P value	0.8814	P value	0.4517	P value	0.502

ROC analysis of baseline CRP levels

CDAI-M3	Results	CDAI-M6	Results	CDAI-M12	Results
Area under the ROC curve	0.5311	Area under the ROC curve	0.6042	Area under the ROC curve	0.5811
Std. Error	0.08147	Std. Error	0.08036	Std. Error	0.08147
95% confidence interval	0.3714 to 0.6908	95% confidence interval	0.4466 to 0.7617	95% confidence interval	0.4214 to 0.7408
P value	0.7042	P value	0.1988	P value	0.3171

SDAI-M3	Results	SDAI-M6	Results	SDAI-M12	Results
Area under the ROC curve	0.5399	Area under the ROC curve	0.6042	Area under the ROC curve	0.5799
Std. Error	0.08144	Std. Error	0.08036	Std. Error	0.08671
95% confidence interval	0.3803 to 0.6996	95% confidence interval	0.4466 to 0.7617	95% confidence interval	0.4099 to 0.7499
P value	0.6278	P value	0.1988	P value	0.3322

ROC analysis of baseline RF

CDAI-M3	Results	CDAI-M6	Results	CDAI-M12	Results
Area under the ROC curve	0.5486	Area under the ROC curve	0.5901	Area under the ROC curve	0.5062
Std. Error	0.08295	Std. Error	0.08329	Std. Error	0.08364
95% confidence interval	0.3860 to 0.7112	95% confidence interval	0.4268 to 0.7533	95% confidence interval	0.3422 to 0.6702
P value	0.5555	P value	0.2722	P value	0.9398

SDAI-M3	Results
Area under the ROC curve	0.5492
Std. Error	0.08342
95% confidence interval	0.3857 to 0.7128
P value	0.553

SDAI-M6	Results
Area under the ROC curve	0.5901
Std. Error	0.08329
95% confidence interval	0.4268 to 0.7533
P value	0.2722

SDAI-M12	Results
Area under the ROC curve	0.527
Std. Error	0.0836
95% confidence interval	0.3631 to 0.6909
P value	0.7449

ROC analysis of baseline anti-CCP

CDAI-M3	Results
Area under the ROC curve	0.5614
Std. Error	0.08155
95% confidence interval	0.4015 to 0.7212
P value	0.4532

CDAI-M6	Results
Area under the ROC curve	0.5699
Std. Error	0.0813
95% confidence interval	0.4106 to 0.7293
P value	0.3883

CDAI-M12	Results
Area under the ROC curve	0.625
Std. Error	0.07828
95% confidence interval	0.4715 to 0.7785
P value	0.1231

SDAI-M3	Results
Area under the ROC curve	0.5584
Std. Error	0.0825
95% confidence interval	0.3966 to 0.7201
P value	0.4785

SDAI-M6	Results
Area under the ROC curve	0.5699
Std. Error	0.0813
95% confidence interval	0.4106 to 0.7293
P value	0.3883

SDAI-M12	Results
Area under the ROC curve	0.6943
Std. Error	0.07244
95% confidence interval	0.5523 to 0.8363
P value	0.0183

anti-CCP, anti-cyclic citrullinated peptide antibodies; CDAI, Clinical Disease Activity Index; CLU, clusterin; CRP, C-reactive protein; DAS28, 28-joint Disease Activity Score; RF, rheumatoid factor; SDAI, Simplified Disease Activity Index
